# Supplementary material for: Variable Induction of Pro-Inflammatory Cytokines by Commercial SARS CoV-2 Spike Protein Reagents: Potential Impacts of LPS on In Vitro Modeling and Pathogenic Mechanisms In Vivo
Source: Int J Mol Sci. 2021 Jul 14;22(14):7540. doi: 10.3390/ijms22147540 (PMC8305765; doi:10.3390/ijms22147540)
Supplement: Supplementary file 1 [file ijms-22-07540-s001.zip › ijms-1274458-supplementary.pdf]

Supplementary Figure S1

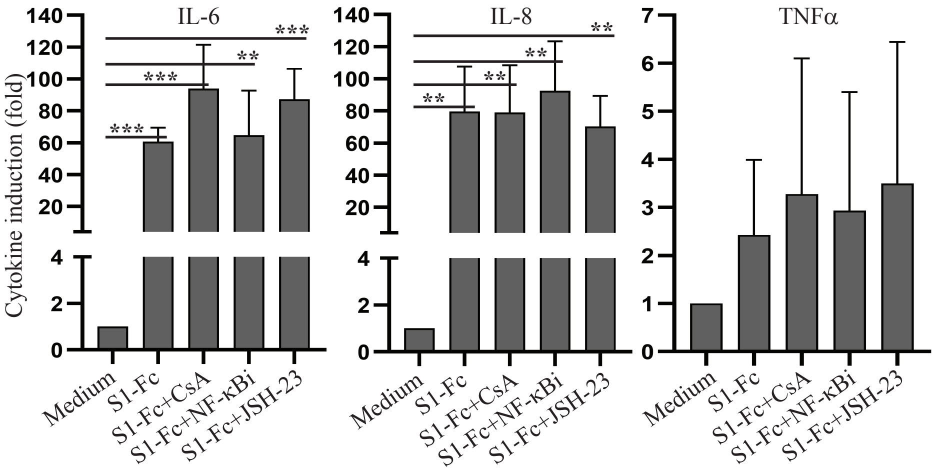

Supplementary Figure S2

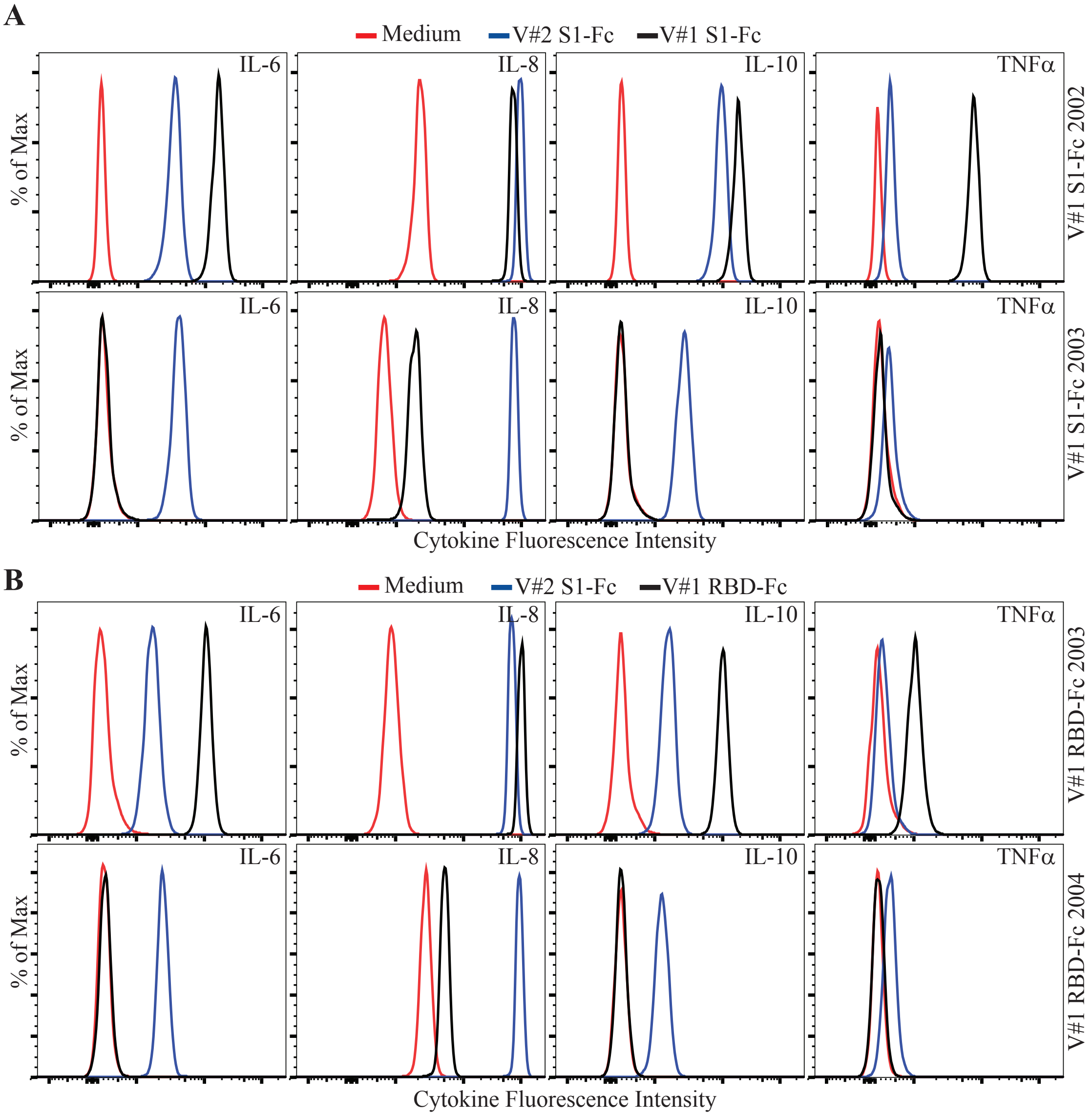

Supplementary Figure S3

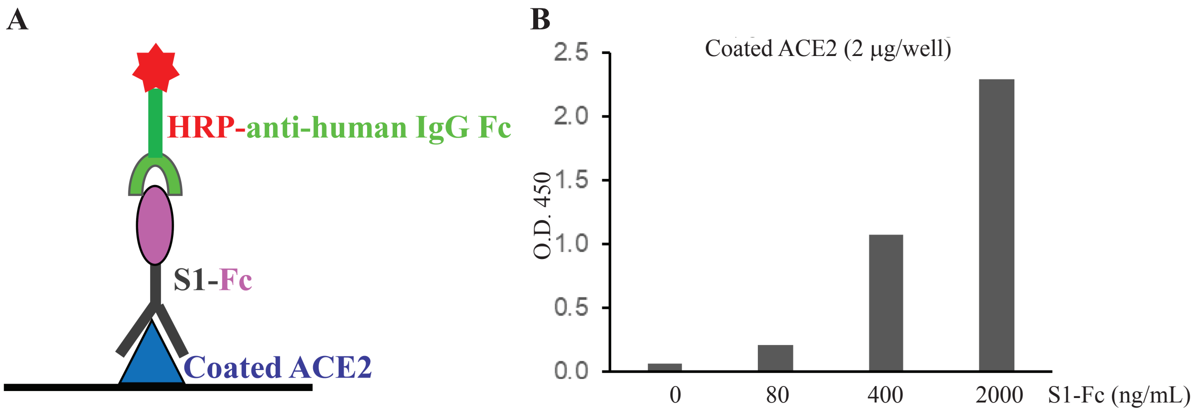

Supplementary Figure S4

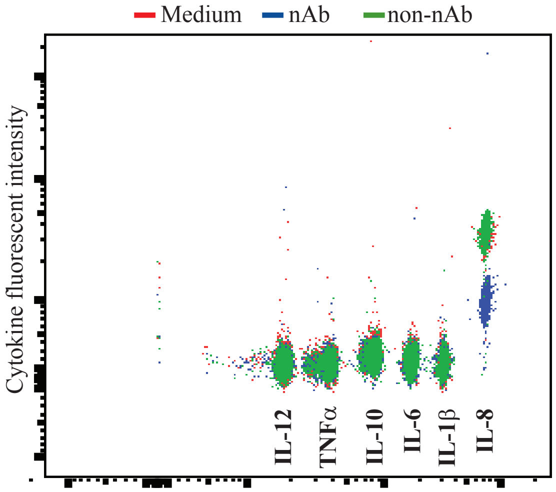

Supplementary Figure S5

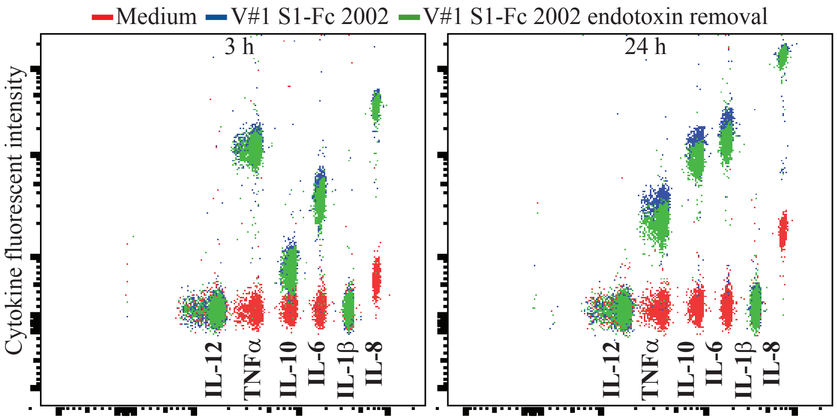

Supplementary Figure S6

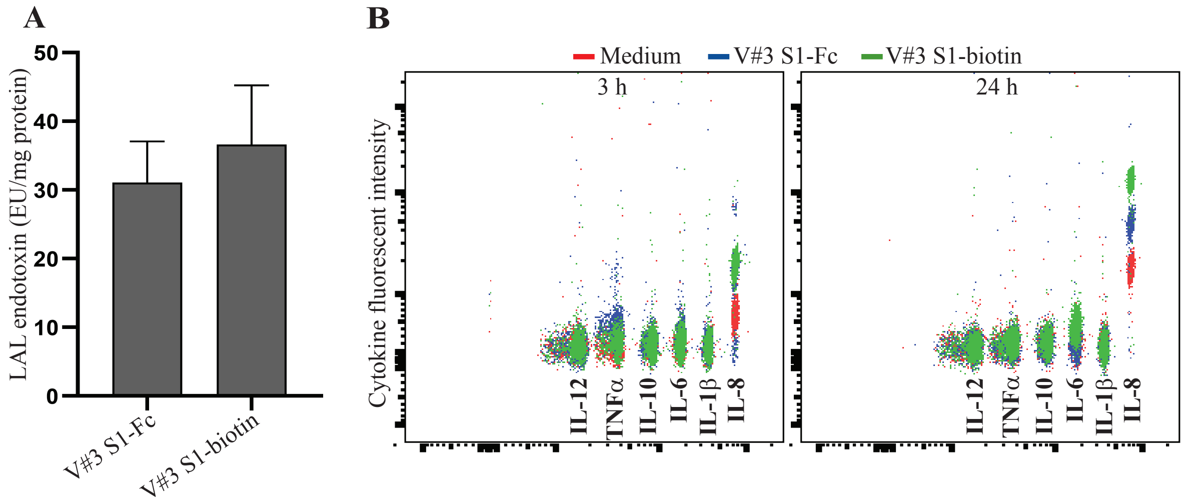

Supplementary Figure S7

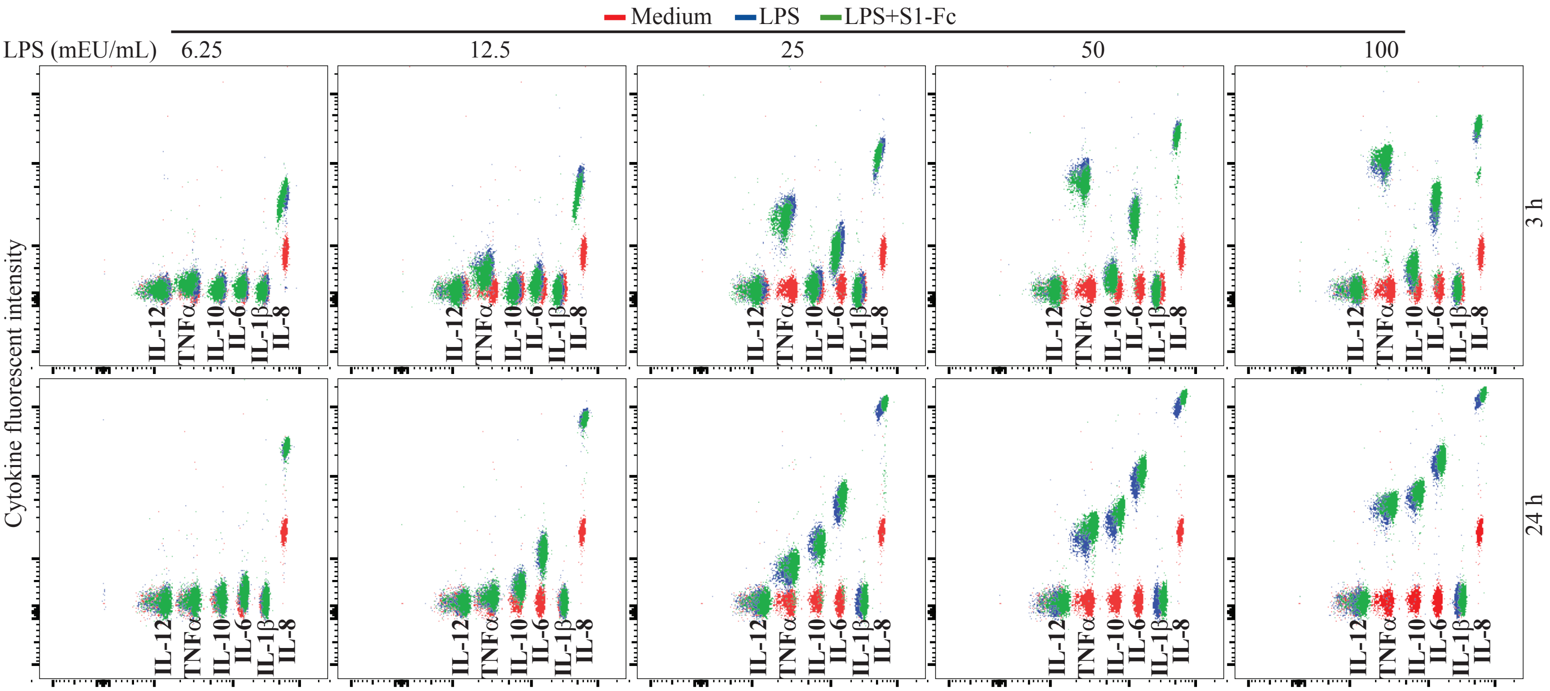

Supplementary Figure S8

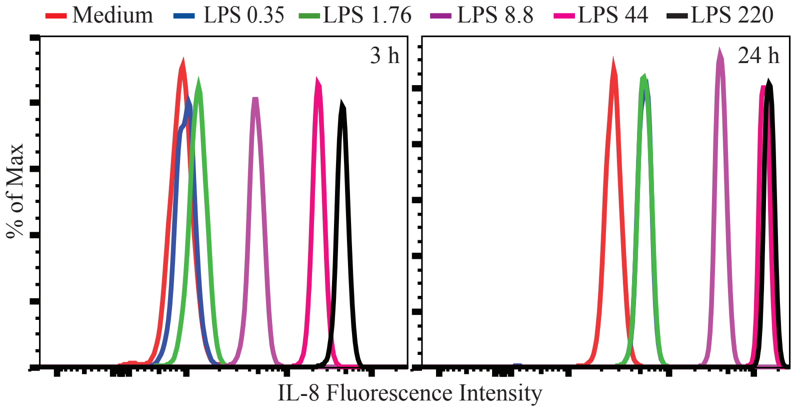

Table S1. Cytokine responses induced by various commercial coronavirus spike proteins

	Cytokine concentration (Mean ± SEM, n=8, pg/mL)		
Treatment	IL-6	IL-8	TNFa
Medium	9.62 ± 0.92	174.69 ± 35.80	33.56 ± 1.32
Raxibacumab	9.52 ± 1.11	141.65 ± 28.46	33.92 ± 1.01
MERS S1-Fc	17.03 ± 6.00	703.85 ± 147.95	35.15 ± 1.46
SARS CoV-1 S1-Fc	9.67 ± 1.18	247.15 ± 37.89	34.37 ± 1.12
V#2 S1-Fc	286.15 ± 89.94	18189.94 ± 6060.44	69.57 ± 20.40
V#2 RBD-Fc	9.85 ± 0.86	359.93 ± 93.84	33.95 ± 0.76
V#1 S1-Fc 2003	11.20 ± 1.37	529.46 ± 106.23	33.93 ± 0.74
V#1 RBD-Fc 2003	3085.08 ± 677.68	34432.54 ± 3501.12	531.00 ± 277.04
S1-Fc+budesonide	22.71 ± 3.16	2387.37 ± 516.27	35.54 ± 1.38
S1-Fc+Erk1/2i	171.89 ± 41.89	5161.93 ± 638.44	35.13 ± 0.96
S1-Fc+JNK1/2i	23.04 ± 4.44	992.50 ± 83.76	32.47 ± 0.78
S1-Fc+p38i	21.79 ± 3.52	4908.09 ± 913.53	31.95 ± 0.56

Table S2 Cytokine responses induced by biotinylated SARS CoV-2 S1 and RBD proteins

	Cytokine concentration (Mean ± SEM, n=3, pg/mL)		
Treatment	IL-6	IL-8	TNFa
medium	12.09 ± 0.90	223.71 ± 29.24	36.38 ± 1.79
S1-biotin/STAV	711.84 ± 179.23	14931.68 ± 349.57	173.23 ± 63.07
RBD-biotin/STAV	34.74 ± 5.90	1610.32 ± 349.57	37.12 ± 1.76
STAV	28.13 ± 3.04	1030.59 ± 121.10	35.60 ± 1.21

Table S3. Polymyxin B inhibition of S1-Fc-induced cytokine responses

	Cytokine concentration (Mean ± SEM, n=3, pg/mL)		
Treatment	IL-6	IL-8	TNFa
medium	9.88 ± 2.95	61.32 ± 21.07	30.96 ± 1.65
S1-Fc	72.81 ± 10.76	1128.14 ± 130.38	148.35 ± 37.45
S1-Fc+polymyxin B	6.51 ± 1.97	209.84 ± 63.53	29.88 ± 0.35

Table S4. SARS CoV-2 spike protein S1 subunit captures endotoxin to induce cytokine responses

	Cytokine concentration (Mean ± SEM, n=4, pg/mL)		
Treatment	IL-6	IL-8	TNFa
LPS 0	6.20 ± 0.49	57.45 ± 10.43	31.20 ± 0.56
LPS 0.1	20.07 ± 5.63	597.54 ± 112.51	66.21 ± 9.24
LPS 0.3	73.28 ± 25.99	1706.10 ± 209.53	180.96 ± 36.41
LPS 0.6	261.42 ± 74.15	3399.15 ± 499.69	642.40 ± 58.91
STAV/LPS 0	5.33 ± 0.46	61.99 ± 12.21	30.49 ± 0.44
STAV/LPS 0.1	19.78 ± 6.06	643.64 ± 82.14	57.37 ± 8.78
STAV/LPS 0.3	97.70 ± 35.24	2139.56 ± 204.22	205.44 ± 41.30
STAV/LPS 0.6	268.58 ± 78.71	3832.19 ± 640.43	589.75 ± 74.94
STAV/S1-Biotin/LPS 0	7.30 ± 1.23	162.26 ± 37.78	34.94 ± 1.07
STAV/S1-Biotin/LPS 0.1	181.23 ± 52.51	2489.02 ± 246.79	253.44 ± 47.13
STAV/S1-Biotin/LPS 0.3	314.15 ± 101.46	4096.00 ± 517.16	577.40 ± 89.92
STAV/S1-Biotin/LPS 0.6	569.98 ± 180.41	5628.75 ± 817.61	1072.73 ± 153.55
